# Sepsis complicated by haemophagocytic lymphohistiocytosis triggered by methicillin-resistant *Staphylococcus aureus* and human herpesvirus 8 in an immunocompromised elderly patient: A case report

**DOI:** 10.1515/biol-2025-1186

**Published:** 2025-10-27

**Authors:** Min He, Yanni Chen, Shanbo Huang, Yongqin Wang, Guanrong Lin, Chaoling Huang

**Affiliations:** Department of Endocrinology, Shishi General Hospital, Fujian Province, China

**Keywords:** sepsis, haemophagocytic syndrome, multiple comorbidities, case report

## Abstract

Sepsis is a life-threatening organ dysfunction caused by a dysregulated host response to infection. A rare but severe complication is haemophagocytic lymphohistiocytosis (HLH), an aggressive syndrome of excessive immune activation. Managing both conditions is particularly challenging in patients with multiple comorbidities. A 78-year-old male with a complex history, including myasthenia gravis and chronic kidney disease, was admitted with sepsis. Investigations confirmed infection with methicillin-resistant *Staphylococcus aureus* and human herpesvirus 8. He was subsequently diagnosed with HLH based on bone marrow findings of haemophagocytosis and elevated soluble CD25 levels. He was treated with a combination of antibiotics, immunomodulatory agents, and supportive care. After 21 days of treatment, the patient’s condition improved significantly. This case highlights the importance of early recognition and timely intervention in the management of sepsis and HLH in patients with multiple comorbidities. A multidisciplinary approach and individualised treatment strategies are crucial for improving patient outcomes.

## 1 Introduction

Sepsis and haemophagocytic lymphohistiocytosis (HLH, also known as haemophagocytic syndrome) are life-threatening critical illnesses in clinical practice [1,2]. When they coexist, the condition becomes complex and the mortality rate increases considerably. The global incidence of sepsis is approximately 48.9 million cases per year, with a mortality rate as high as 20–30%. This is particularly true among elderly patients, where immunosenescence and underlying diseases further exacerbate poor prognosis [3,4]. As an excessive inflammatory response syndrome, HLH is often triggered by infections, tumours, or autoimmune diseases. Its pathophysiological mechanisms involve cytokine storms and abnormal macrophage activation. Diagnostic criteria include fever, cytopenia, hyperserotonaemia, and haemophagocytosis in the bone marrow [5,6]. Recent studies have shown that mixed bacterial and viral infections, such as methicillin-resistant *S. aureus* (MRSA) combined with human herpesvirus 8 (HHV-8), can synergistically activate toll-like receptors (TLRs) and interferon pathways, accelerating the progression of HLH [7]. The interplay between pathogens can be particularly potent. This synergistic activation can create a “cytokine storm” that overwhelms regulatory mechanisms, thus accelerating the progression to fulminant HLH.

The considerable overlap in clinical and laboratory features between severe sepsis and secondary HLH creates a significant diagnostic dilemma for clinicians. For example, both conditions present with fever, cytopenias, and hyperferritinemia. This mimicry is particularly challenging because their management strategies diverge critically: while sepsis requires aggressive antimicrobial therapy and supportive care, HLH necessitates prompt immunomodulatory or cytotoxic treatment to control hyperinflammation. This creates a high-stakes clinical scenario, where the delayed diagnosis of HLH or the premature use of potent immunosuppression in a patient with uncontrolled infection can lead to catastrophic outcomes. This challenge is further magnified in elderly patients with multiple comorbidities, where immune senescence and underlying organ dysfunction limit therapeutic options and increase the risk of adverse events.

Infectious triggers of secondary HLH include bacteria (notably staphylococci and streptococci) and herpesviruses [8]; among the latter, HHV-8 (also known as Kaposi sarcoma herpesvirus) is a recognised cause of severe inflammatory syndromes in immunocompromised hosts. In addition, chronic diseases such as hypertensive heart disease, diabetes, and chronic kidney disease not only increase the risk of sepsis but also limit the choice of antimicrobial drugs and immunomodulatory treatments [9]. Diagnostic aids, such as the HScore, and molecular tests, such as metagenomic next-generation sequencing (mNGS), have recently been used to identify multi-pathogen triggers and inform management in complex cases [10].

This study discusses an elderly patient with multiple comorbidities who developed MRSA bacteraemia with concurrent HHV-8 viremia, resulting in secondary HLH. The case highlights diagnostic and therapeutic dilemmas, including integration of culture and mNGS results, decision-making about immunomodulation in the context of active bacteraemia, and tailored post-discharge follow-up in a frail patient. Our objective is to present the diagnostic reasoning and management decisions that may inform care for similar high-risk patients.

Therefore, this case report aims to illustrate the diagnostic and therapeutic complexities encountered in an elderly, immunocompromised patient with sepsis-triggered HLH due to a dual bacterial-viral infection. We highlight the challenges of (1) integrating novel diagnostic results like mNGS with traditional culture methods, (2) carefully titrating immunomodulatory therapy in the setting of active bacteremia, and (3) developing a tailored, long-term follow-up plan for a frail patient. Through this case, we aim to provide insights that may aid clinicians in managing this high-risk and challenging patient population.

## 2 Case presentation

### 2.1 Patient information

#### 2.1.1 Patient profile

A 78-year-old man with a history of grade 2 hypertension, hypertensive heart disease (New York Heart Association Class II), chronic kidney disease (Stage 3), myasthenia gravis, and thymoma was admitted to our hospital on 24 December 2022, with a chief complaint of chills and fever for 1 day. His past medical history was notable for multiple prior hospitalisations due to pneumonia and sepsis, and he was on long-term immunosuppressive therapy with oral prednisone 10 mg twice daily and mycophenolate mofetil 0.5 g twice daily for myasthenia gravis. One month prior to the current admission, he had been hospitalised for MRSA-related pneumonia and sepsis. He did not meet the criteria for HLH at that time and was discharged with a 14-day course of oral linezolid (600 mg twice daily).


**Informed consent:** Informed consent has been obtained from all individuals included in this study.
**Ethical approval:** The research related to human use has been complied with all the relevant national regulations, institutional policies, and in accordance with the tenets of the Helsinki Declaration, and has been approved by the Ethics Committee of Shishi General Hospital.

#### 2.1.2 Chief complaints and admission history

The patient presented with persistent high fever (maximum temperature 39.5°C), cough, expectoration of yellow purulent sputum, dyspnoea on exertion, and worsening of a skin ulcer on the left lower limb. One month prior to admission, he was hospitalised for “pulmonary infection and sepsis (*S. aureus*).” After discharge, he regularly took linezolid tablets, steroids, and immunomodulatory drugs. Three days before this admission, he developed high fever and dyspnoea, with progressive thrombocytopenia (72 → 40 × 10^9^/L). Three days prior to admission, he experienced coughing (mild, single coughs, worse at night, mainly dry cough) with chest discomfort, dyspnoea on exertion (relieved by rest), and bilateral lower limb oedema. He did not report chills, fever, sore throat, nasal congestion, rhinorrhoea, headache, myalgia, palpitations, oliguria, facial oedema, abdominal pain, bloating, vomiting, dizziness, or fatigue. His symptoms recurred, and he self-measured a temperature of 37.4°C with peripheral oxygen saturation of 80%. He sought further diagnosis and treatment at the hospital.

#### 2.1.3 Admission examination

**Figure 1:**
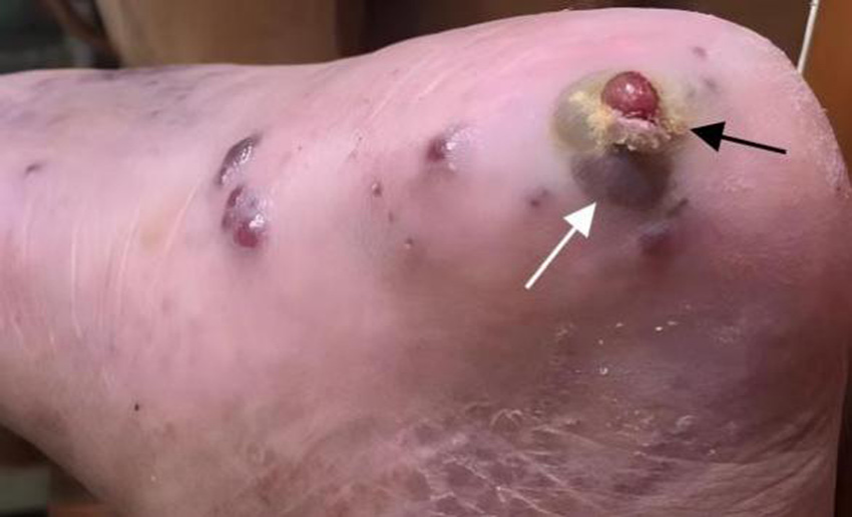
Skin ulcer of 3 cm × 5 cm was found in the left lower limb, with basal necrosis and purulent secretion. The white arrow indicates basal necrosis, and the black arrow indicates purulent secretion.

On admission, his temperature was 37.5°C, heart rate 85 bpm, respiratory rate 20/min, blood pressure 105/64 mmHg, and oxygen saturation 96% with 50% oxygen via face mask. Physical examination revealed bilateral crackles on lung auscultation, a 3 cm × 5 cm necrotic ulcer with purulent discharge on the left lower limb, and an uninfected sacral wound (Figure 1). Laboratory evaluation (Table 1) revealed leukopenia and progressive thrombocytopenia (platelets 40 × 10⁹/L, haemoglobin 111 g/L), elevated inflammatory markers (C-reactive protein [CRP] 128.53 mg/L, procalcitonin [PCT] 27.78 ng/mL, erythrocyte sedimentation rate 115 mm/h), hypoalbuminemia (24 g/L), hyponatremia (126.1 mmol/L), and fasting hyperglycaemia (15.4 mmol/L). Blood cultures yielded MRSA, and HHV-8 was detected in peripheral blood by PCR (92.29%). Bone marrow aspiration demonstrated haemophagocytosis (2.8%) and markedly elevated serum soluble CD25 (sCD25) (14,227 U/mL). Although serum ferritin and triglyceride values were not initially available, the patient fulfilled at least five HLH-2004 diagnostic criteria, supporting the diagnosis of secondary HLH. Chest computed tomography (CT) showed bilateral patchy consolidations and moderate pleural effusions. Echocardiography indicated mildly elevated pulmonary artery systolic pressure (estimated 44 mmHg) with preserved left ventricular ejection fraction. The pleural effusion was presumed to be due to inflammation. No ascites or pericardial effusion was noted.

**Table 1:** Changes in key clinical indicators from admission to discharge

Category	On admission	At discharge	Notes
**Vital signs**			
Temperature (°C)	37.5	Normal	Temperature returned to normal
Heart rate (bpm)	85	Normal	Heart rate returned to normal
Respiratory rate (bpm)	20	Normal	Respiratory rate returned to normal
Blood pressure (mmHg)	105/64	Stable	Blood pressure stabilised
SpO_2_ (%)	96 (supplemental oxygen)	Normal	Blood oxygen saturation returned to normal
**Inflammatory indicators**			
CRP (mg/L)	128.53	3.43	Significant decrease
PCT (ng/mL)	27.78	0.16	Significant decrease
ESR (mm/h)	115	Normal	Significant decrease
**Laboratory tests**			
Platelets (×10^9^/L)	40	545	Increase
Haemoglobin (g/L)	111	Normal	Increased to the normal range
Albumin (g/L)	24	>30	Supplemented to the normal target range
Sodium (mmol/L)	126.1	Normal	Corrected to the normal range
Fasting plasma glucose (mmol/L)	15.4	<10	Significant decrease
**Imaging**			
Chest CT	Patchy consolidation with pleural effusion	70% absorption of infectious foci and pleural effusion is basically absorbed	Significant improvement
Echocardiography	Elevated PA pressure (44 mmHg)	Maintained control	Maintained control of PA pressure

“Normal” refers to values within the institutional reference range at our center.

#### 2.1.4 Key diagnoses

The patient was diagnosed with sepsis, presenting with a mixed infection involving MRSA bacteria and HHV-8, meeting the criteria for systemic inflammatory response syndrome. Furthermore, the patient fulfilled the HLH-2004 diagnostic criteria, exhibiting fever, cytopenia, haemophagocytosis in the bone marrow, and elevated sCD25 levels, confirming HLH. Additionally, the patient had multiple comorbidities, including pulmonary infection (confirmed by imaging and aetiology), steroid-induced diabetes (fasting blood glucose 15.4 mmol/L), thrombocytopenia, moderate anaemia, hypoproteinaemia, myasthenia gravis, Grade 2 hypertension (very high risk), hypertensive nephropathy, atherosclerosis of the aorta and coronary arteries, and multiple pulmonary nodules.

#### 2.1.5 Treatment course

Antimicrobial therapy was initiated with intravenous meropenem 1 g every 12 h and vancomycin 1 g every 12 h, which was later narrowed to vancomycin monotherapy for 14 days based on culture sensitivity. Ganciclovir 350 mg every 12 h was administered intravenously to target HHV-8 viremia. Immunomodulatory treatment included intravenous methylprednisolone at 40 mg once daily, tapered to 24 mg daily over 10 days, and intravenous immunoglobulin (IVIG) 20 g daily for 5 consecutive days. Supportive care included platelet transfusions for counts <20 × 10^9^/L, red blood cell transfusions for symptomatic anaemia, continuous insulin infusion to control hyperglycaemia (target fasting glucose <10 mmol/L), and intravenous albumin supplementation to maintain serum albumin >30 g/L. Loop diuretics (torasemide) were used to control fluid balance and pulmonary congestion.

#### 2.1.6 Outcome

After 21 days of treatment, the patient’s temperature normalised, inflammatory markers significantly decreased (CRP 3.43 mg/L, PCT 0.16 ng/mL), and platelets increased to 545 × 10^9^/L. Chest CT showed 70% absorption of the infectious foci. At discharge, he occasionally coughed but had no fever or dyspnoea, with stable blood glucose and blood pressure. Regular follow-up of blood routine, inflammatory markers, and imaging changes was recommended after discharge. The patient’s inflammatory markers gradually decreased, blood cells increased, and physiological indicators stabilised. At discharge, his cough and dyspnoea were significantly relieved, with no fever, mild fatigue, improved mental status, appetite and sleep, normal bowel movements, no oliguria, stable weight, and normal urinalysis. The patient’s condition was stable, and he was discharged in good general condition.

#### 2.1.7 Follow-up and prognosis

The patient was advised to return for structured outpatient follow-up given his advanced age, multiple comorbidities, and recent immunosuppressive therapy. A practical monitoring plan was arranged as follows: (1) a telephone check within 7 days after discharge to review symptoms and medication adherence; (2) outpatient clinic visits at 2–4 weeks and 8–12 weeks after discharge, then every 3 months up to 1 year or as clinically indicated; (3) laboratory monitoring with complete blood count and basic metabolic panel weekly until haematologic recovery, then every 2–4 weeks until 3 months and thereafter at each clinic visit; inflammatory markers (CRP, PCT as appropriate), serum ferritin and liver/renal function tests at each visit; (4) targeted monitoring of HHV-8 viral load (if available) and sCD25/ferritin in the early post-discharge period (monthly for the first 3 months) to detect reactivation or relapse; and (5) early re-presentation if fever, new cytopenia, dyspnoea or wound deterioration occurs. Follow-up priorities included infection surveillance, glucose control, medication reconciliation (especially immunosuppressants/antibiotics), and functional assessment/rehabilitation planning. The schedule was individualised and could be intensified according to clinical course.

## 3 Discussion

This case involves a 78-year-old male patient with comorbidities, including hypertensive heart disease, myasthenia gravis, and thymoma. He developed sepsis complicated by HLH due to a mixed infection of MRSA and HHV-8. The diagnosis and treatment process highlighted the complexity of the interplay between immune dysfunction and infection in elderly patients with multiple comorbidities. The following analysis is presented from three aspects: pathophysiological mechanisms, therapeutic contradictions, and clinical insights.

### 3.1 Ageing and immune deficiency: The vicious cycle of infection and HLH

Advanced age and comorbidities are associated with immune senescence and increased risk of both severe bacterial infection and viral reactivation. In this patient, bacteraemia with MRSA likely provided a potent pro-inflammatory stimulus, and concurrent HHV-8 viremia may have acted as a second hit, amplifying immune activation [11]. In this case, bone marrow examination revealed haemophagocytic cells accounting for 2.8%, with significantly elevated sCD25 (14,227 U/mL), meeting the diagnostic criteria for HLH. The markedly elevated and sCD25 alongside progressive cytopenia are consistent with cytokine-mediated haemophagocytic activity and informed our decision to institute cautious immunomodulation (low-to-moderate dose corticosteroids with close infection surveillance), rather than aggressive cytotoxic therapy. Moreover, recent research has shown that HHV-8 can activate macrophages via the TLR3/4 pathway and induce excessive secretion of interferon gamma (IFN-γ), further amplifying the inflammatory cascade of HLH [12].

### 3.2 Pathogenesis: Ageing, immune senescence, and mixed infectious triggers

The mortality rate increases considerably in elderly patients with mixed bacterial and viral infections. In this case, blood culture detected MRSA, and mNGS suggested HHV-8 infection. The synergistic effect of the two exacerbated immune dysfunction. Studies have shown that superantigens secreted by *S. aureus* (such as toxic shock syndrome toxin-1) can non-specifically activate T cells, driving the progression of HLH, together with IFN-γ release mediated by the virus [13]. However, there were multiple contradictions in the treatment – broad-spectrum antibiotics (such as vancomycin) could control bacterial infections but may worsen kidney damage; glucocorticoids could suppress cytokine storms but increase the risk of blood glucose fluctuations and infection spread. In this case, meropenem combined with vancomycin was used to cover MRSA. The choice of a moderate initial dose of methylprednisolone (40 mg daily) was a deliberate strategy to balance the suppression of the HLH-associated hyperinflammation against the significant risk of exacerbating the underlying bacterial infection in a frail, elderly patient [14]. The subsequent tapering to 24 mg daily was not based on a fixed schedule but was dynamically guided by close monitoring of the patient’s clinical and laboratory response. Specifically, the decision to taper was made after observing a consistent normalisation of temperature for over 72 h and a sharp decline in inflammatory markers, particularly C-reactive protein (CRP) and procalcitonin (PCT). This approach allowed for an individualised treatment that minimised the total steroid exposure while ensuring the inflammatory process was adequately controlled. The choice was made empirically because antivirals such as ganciclovir/valganciclovir have been shown to suppress HHV-8 replication and have been used in HHV-8-associated conditions (e.g. Kaposi sarcoma and multicentric Castleman disease) and in case reports of HHV-8-associated hyperinflammation; however, randomised evidence for clear clinical benefit in HHV-8-associated HLH is lacking. We therefore used antiviral therapy as adjunctive care while prioritising targeted antibiotics and immunomodulatory treatment [15].

### 3.3 Treatment challenges: Balancing immunosuppression and infection control

Understanding these mechanisms clarified the therapeutic priorities, namely suppressing deleterious hyperinflammation while preserving anti-infective defences. Elderly patients with multiple comorbidities require multidisciplinary collaboration to optimise treatment. In this case, the patient had comorbid hypertensive heart disease and steroid-induced diabetes, limiting fluid resuscitation and hormone dosage. Research indicated that dynamic monitoring of blood volume (such as echocardiography) and strict blood glucose control (target range 7.8–10 mmol/L) could improve prognosis [16]. Additionally, the use of IVIG needed to weigh cost against benefit; in this case, IVIG 20 g × 5 days was administered, which increased the platelet count in the short term. However, a recent meta-analysis suggested that IVIG had limited improvement on long-term survival rate in secondary HLH, recommending its use only in severe or refractory cases [17].

### 3.4 Literature comparison and clinical insights

In this case, plasma mNGS identified HHV-8 viremia that was not detected by routine culture methods. As mNGS can broaden pathogen detection in complex cases, we treated the mNGS HHV-8 result as supportive evidence, prompting confirmatory testing. Recent literature has highlighted several issues relevant to our case. First, HHV-8 reactivation is an established trigger of severe inflammatory syndromes in immunosuppressed hosts; antiviral agents, such as ganciclovir/valganciclovir, reduce HHV-8 replication and have been used in Kaposi sarcoma and multicentric Castleman disease, although high-quality evidence for HHV-8-related HLH is limited [18]. Therefore, antiviral therapy may be considered as adjunctive management in cases with documented high viral load, particularly in immunocompromised patients. Second, adult HLH management is evolving; beyond the HLH-94/2004 paradigms, newer targeted approaches (e.g. JAK inhibitors, such as ruxolitinib, and cytokine-targeting agents, such as emapalumab) show promise in refractory or relapsed secondary HLH and are subjects of active study [19]. However, evidence in elderly patients and the setting of active bacterial sepsis remains limited and should be weighed against infection risk and drug access/cost. Third, elderly sepsis survivors are at high risk of early readmission, functional decline, and mortality; therefore, in this case, etoposide was not used, and the condition was stabilised through enhanced supportive treatment (platelet transfusion and albumin supplementation), providing an alternative strategy for frail elderly patients [20].

### 3.5 Long-term prognosis and follow-up limitations

Despite the patient’s successful recovery and discharge, it is crucial to acknowledge that his long-term prognosis remains guarded. Survivors of sepsis and HLH, particularly the elderly with multiple comorbidities, are at a high risk of post-sepsis syndrome, characterised by functional decline, cognitive impairment, and increased susceptibility to future infections. Moreover, the underlying myasthenia gravis necessitates ongoing immunosuppression, which perpetually increases the risk of both HLH relapse and recurrent sepsis. Our proposed follow-up plan, while structured, has inherent limitations. The practical feasibility of frequent laboratory monitoring, including specialised tests like sCD25 and HHV-8 viral load, can be challenging in an outpatient setting due to cost and accessibility. Furthermore, patient adherence to such a rigorous schedule can be variable. This underscores the need for a highly vigilant, multidisciplinary approach to post-discharge care, with a low threshold for re-evaluation if any new symptoms arise.

## 4 Conclusion

This case highlights the diagnostic and therapeutic complexities when sepsis and HLH coexist in an elderly patient with multiple comorbidities and prior immunosuppression. Early recognition, targeted antimicrobial therapy, and multidisciplinary management remain central to favourable outcomes. Beyond these points, the case underscores three broader implications: (1) in immunocompromised elderly adults, clinicians should consider combined infectious triggers (bacterial and viral) and, where available, include viral load monitoring to guide adjunctive antiviral therapy; (2) immunomodulatory strategies must be individualised – balancing control of hyperinflammation and infection risk – and close post-discharge surveillance is essential to detect relapse or complications; and (3) further research into rapid diagnostics, biomarkers (e.g. dynamic ferritin and sCD25) and age-appropriate therapeutic algorithms (including investigation of targeted agents, such as JAK inhibitors or IFN-γ antagonists in refractory cases) is needed to improve outcomes in this high-risk population.
